# Synthesis and structure of 5-[(4-fluoro­benz­yl)amino]­isophthalic acid featuring O—H⋯O and N—H⋯π inter­actions

**DOI:** 10.1107/S205698902600959X

**Published:** 2026-09-25

**Authors:** Atash V. Gurbanov, Mehmet Akkurt, Anirban Karmakar, Gizachew Mulugeta Manahelohe

**Affiliations:** a Excellence Center, Baku State University, Z. Khalilov Str. 33, AZ 1148, Baku, Azerbaijan; b Department of Physics, Faculty of Sciences, Erciyes University, 38039 Kayseri, Türkiye; c Centro de Estudos de Engenharia Química, Instituto Superior de Engenharia de Lisboa, Instituto Politécnico de Lisboa, 1959-007 Lisboa, Portugal; d Department of Chemistry, University of Gondar, PO Box 196, Gondar, Ethiopia; University of Aberdeen, United Kingdom

**Keywords:** crystal structure, carb­oxy­lic acid homodimers, Hirshfeld surface analysis

## Abstract

In the title compound, C_15_H_12_FNO_4_, the dihedral angle between the aromatic rings is 77.78 (8)°. In the crystal, the mol­ecules are linked into zigzag ribbons propagating along the [210] direction by pairwise O—H⋯O hydrogen bonds through their two carb­oxy­lic acid groups to generate centrosymmetric *R*^2^_2_(8) dimers in each case. In addition, mol­ecules are connected *via* pairwise N—H⋯π inter­actions between the NH groups and the fluoro­benzene rings. Aromatic π–π stacking inter­actions and weak C—H⋯F and C—H⋯O inter­actions also contribute to the packing. A Hirshfeld surface analysis shows that the most important contributions to the crystal packing are from H⋯H (30.7%), O⋯H/H⋯O (26.1%) and C⋯H/H⋯C (20.3%) contacts.

## Abstract 2

In the title compound, the dihedral angle between the aromatic rings is 77.78 (8)°. In the crystal, the mol­ecules are linked into zigzag ribbons propagating along the [210] direction by pairwise O—H⋯O hydrogen bonds through their two carb­oxy­lic acid groups to generate centrosymmetric *R*^2^_2_(8) dimers in each case. In addition, mol­ecules are connected *via* pairwise N—H⋯π inter­actions between the NH groups and the fluoro­benzene rings.

## 1. Chemical context

Metal–organic frameworks (MOFs) are highly porous crystalline materials built from metal ions connected by organic ligands (Chughtai *et al.*, 2015 ▸; Liu *et al.*, 2014 ▸). The functional properties of MOFs can be improved by the design of organic ligands (carboxyl­ates, sulfonates, phospho­nates, *etc*.) and the variation of metal centres. 5-Amino­isophthalic acid and its derivatives have already been used in the synthesis and design of MOFs (Karmakar *et al.*, 2024 ▸). In order to generate an additional coordination centre and a non-covalent bond donor or acceptor site, 5-amino­isophthalic acid has been functionalized by introducing hydrazone, amide, azo, *etc*. onto its backbone (Aliyeva *et al.*, 2026 ▸). For example, halogen-bonded hydrazones of 5-amino-2,4,6-tri­bromo­isophthalic acid were employed in the construction of new MOFs and applied in the Henry reaction of aldehydes with nitro­alkanes (Gurbanov *et al.*, 2022 ▸) as well as in the coupling of CO_2_ with epoxides (Aliyeva *et al.*, 2023 ▸). As a continuation of our research work in this direction, we now describe the synthesis and structure of the title compound, C_15_H_12_FNO_4_ (**I**).
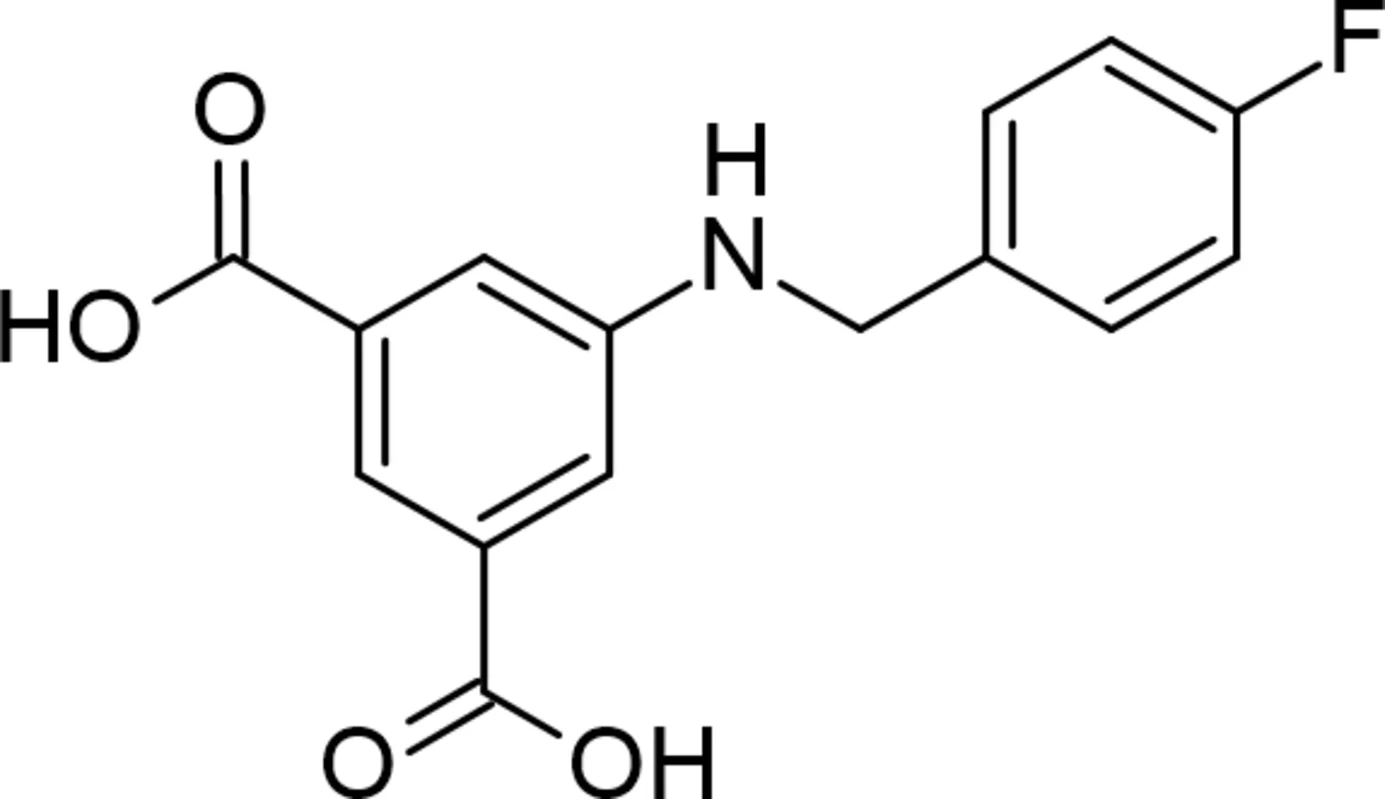


## 2. Structural commentary

Compound (**I**) crystallizes in the triclinic space group *P*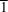 with one mol­ecule in the asymmetric unit (Fig. 1 ▸). The dihedral angle between the planes of the benzene-1,3-di­carb­oxy­lic acid (C1–C6) and fluoro­phenyl (C8–C13) rings is 77.78 (8)°. The sum of the bond angles around N1 is 358.7°, implying *sp*^2^ hybridization; the unhybridized 2*p* orbital is well aligned to inter­act with the π system of the C1–C6 ring, which may result in partial double-bond character for the C1—N1 bond [1.3728 (19) Å]. The C14/O1/O2 and C15/O3/O4 carb­oxy­lic acid groups are almost coplanar with their attached ring [dihedral angles = 3.83 (19) and 6.7 (3)°, respectively].

The C8—C7—N1 bond angle of 115.64 (13)° for the bridging methyl­ene group deviates significantly from the nominal tetra­hedral value of 109.5° possibly due to steric crowding. The torsion angles of the C2—C1—N1—C7, C1—N1—C7—C8 and N1—C7—C8—C13 groupings are 164.21 (15), 89.38 (19) and 173.68 (14)°, respectively. A short intra­molecular C—H⋯N contact (Table 1 ▸) is observed. Otherwise, all geometric parameters for (**I**) are normal and consistent with those of related compounds listed in the *Database survey*.

## 3. Supra­molecular features and Hirshfeld surface analysis

In the extended structure of (**I**), the mol­ecules are connected into zigzag ribbons propagating along the [210] direction by two centrosymmetric 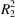
(8) dimers formed from pairwise O—H⋯O hydrogen bonds involving both their carb­oxy­lic acid groups (Fig. 2 ▸, Table 1 ▸). Furthermore, mol­ecules are linked by pairwise N—H⋯π inter­actions between the NH groups and fluoro­benzene rings (Fig. 3 ▸, Table 1 ▸). Simultaneously, the mol­ecules are linked by weak π–π stacking inter­actions [*Cg*1⋯*Cg*1^*a*^ = 3.9798 (10) Å, shift = 2.081 Å; symmetry code (*a*) 1 − *x*, 1 − *y*, −*z*; *Cg*1 is the centroid of the C1–C6 benzene ring], generating ribbons propagating along the [011] direction (Fig. 3 ▸, Table 1 ▸). Finally, weak C—H⋯F and C—H⋯O inter­actions between these ribbons may help to consolidate the packing.

A Hirshfeld surface analysis was carried out to further investigate the inter­molecular inter­actions using *Crystal Explorer 21* (Spackman *et al.*, 2021 ▸). The Hirshfeld surface mapped over *d*_norm_ is illustrated in Fig. 4 ▸. The red spots on the Hirshfeld surface plot indicate the directional inter­actions noted above. The two-dimensional fingerprint plots are shown in Fig. 5 ▸: H⋯H (30.7%), O⋯H/H⋯O (26.1%) and C⋯H/H⋯C (20.3%) contacts are the main contributors to the crystal packing. Minor contributions are from F⋯H/H⋯F (7.5%), O⋯C/C⋯O (4.4%), C⋯C (4.0%), F⋯C/C⋯F (2.7%), F⋯O/O⋯F (2.3%), O⋯O (1.1%), N⋯C/C⋯N (0.6%) and F⋯F (0.3%) contacts.

## 4. Database survey

A search of the Cambridge Structural Database (CSD, Version 6.00, update of April 2025; Groom *et al.*, 2016 ▸) found that the most closely related structures to the title compound are *N*-benzyl­aniline (CSD refcode UREJOR: Betz *et al.*, 2011 ▸), *N*-benzyl-3-nitro­aniline (YAHDIW: Stilinović & Portada, 2011 ▸) and *N*-[3,5-bis­(tri­fluoro­meth­yl)phen­yl]-1-(2-bromo­phen­yl)methanimine (AKISIA: Kingsley *et al.*, 2025 ▸).

In UREJOR, the N atom is present in a nearly planar mol­ecular geometry (angle sums at the N atom are 358 and 359° in the two mol­ecules of the asymmetric unit). The planes defined by the aromatic rings inter­sect at angles of 80.76 (4) and 81.40 (4)° in the two mol­ecules. In the crystal, N—H⋯π inter­actions connect the two mol­ecules of the asymmetric unit to form infinite homodromic chains propagating along the *b* axis direction [N⋯π = 3.4782 (12) and 3.4642 (13) Å].

In YAHDIW, the *N*–meth­yl–3–nitro­aniline system is almost planar and the dihedral angle between the ring planes is 86.0 (6)°. In the crystal, the mol­ecules are connected *via* strong N—H⋯O and weak C—H⋯O hydrogen bonds into chains propagating along [101].

In AKISIA, the imine grouping is nearly coplanar with the 2-bromo­phenyl group, while the *N*-[3,5-bis­(tri­fluoro­meth­yl)phen­yl] moiety is significantly twisted from the imine group [49.61 (5)°]. The imine bond length C7—N1 [1.280 (4) Å] and C7—N1—C8 bond angle [119.1 (2)°] are consistent with atom N1 being *sp*^2^ hybridized as in (**I**). The angle between the least-squares planes formed by C1–C8/N1/Br1 and C8–C15/N1 of 49.61 (5)° is consistent with steric effects involving H7 and H9. The crystal packing involves short C—H⋯Br contacts generating zigzag ribbons flanked by fluorine-rich layers.

## 5. Synthesis and crystallization

A mixture of 5-amino­isophthalic acid (1.08 g, 6 mmol) and 4-fluoro­benzaldehyde (0.992 mg, 8.0 mmol), in ethanol (95%, 30 ml) was stirred at room temperature. Tri­ethyl­amine (4.16 ml, 30 mmol) was added to the mixture and stirred for 1 h. The mixture became viscous, then an excess of NaBH_4_ (2 equivalents relative to the imine) was slowly added at 273 K. After 4 h at 273 K, the solvent was removed under vacuum. The residue was dissolved in water (50 ml) and acidified with glacial acetic acid (AcOH) to pH 3–5. After filtration, the product was obtained as a pale-yellowish crystalline solid. Single crystals of (I) were recrystallized from a methanol solution at room temperature. Yield: 71%. Analysis calculated for C_15_H_12_FNO_4_: C 62.28; H 4.18; N 4.84; found: C 62.26; H 4.15; N 4.81%. ^1^H NMR (DMSO-*d*_6_) δ 13.20 (*br s*, 2H, –COOH), 7.81 (*s*, 1H, Ar), 7.76 (*s*, 2H, Ar), 7.33 and 7.30 (*m*, 2H, *J* = 12.0 Hz, Ar), 6.96 and 6.93 (*m*, 2H, *J* = 12.0 Hz, Ar), 6.75 (*s*, 1H, NH), 4.62 (*d*, 2H, CH_2_). ^13^C NMR (DMSO-*d*_6_) δ 161.2, 161.1, 156.7, 144.1, 139.0, 129.2, 127.2, 127.1, 115.3, 113.4, 46.7.

## 6. Refinement

Crystal data, data collection and structure refinement details are summarized in Table 2 ▸. All hydrogen atoms were found from difference-Fourier maps. Hydroxyl group H atoms wererefined with *U*_iso_(H) = 1.5*U*_eq_(O), while other H atoms were refined freely. The restraints O—H = 0.85 (1) Å for the OH group, N—H = 0.86 (1) Å for the NH group, C—H = 0.99 Å for the CH_2_ group, and C—H = 0.95 Å for the CH group were applied using the DFIX command.

## Supplementary Material

Crystal structure: contains datablock(s) I. DOI: 10.1107/S205698902600959X/hb8253sup1.cif

Structure factors: contains datablock(s) I. DOI: 10.1107/S205698902600959X/hb8253Isup2.hkl

Supporting information file. DOI: 10.1107/S205698902600959X/hb8253Isup3.cml

CCDC reference: 2587990

Additional supporting information:  crystallographic information; 3D view; checkCIF report

## Figures and Tables

**Figure 1:**
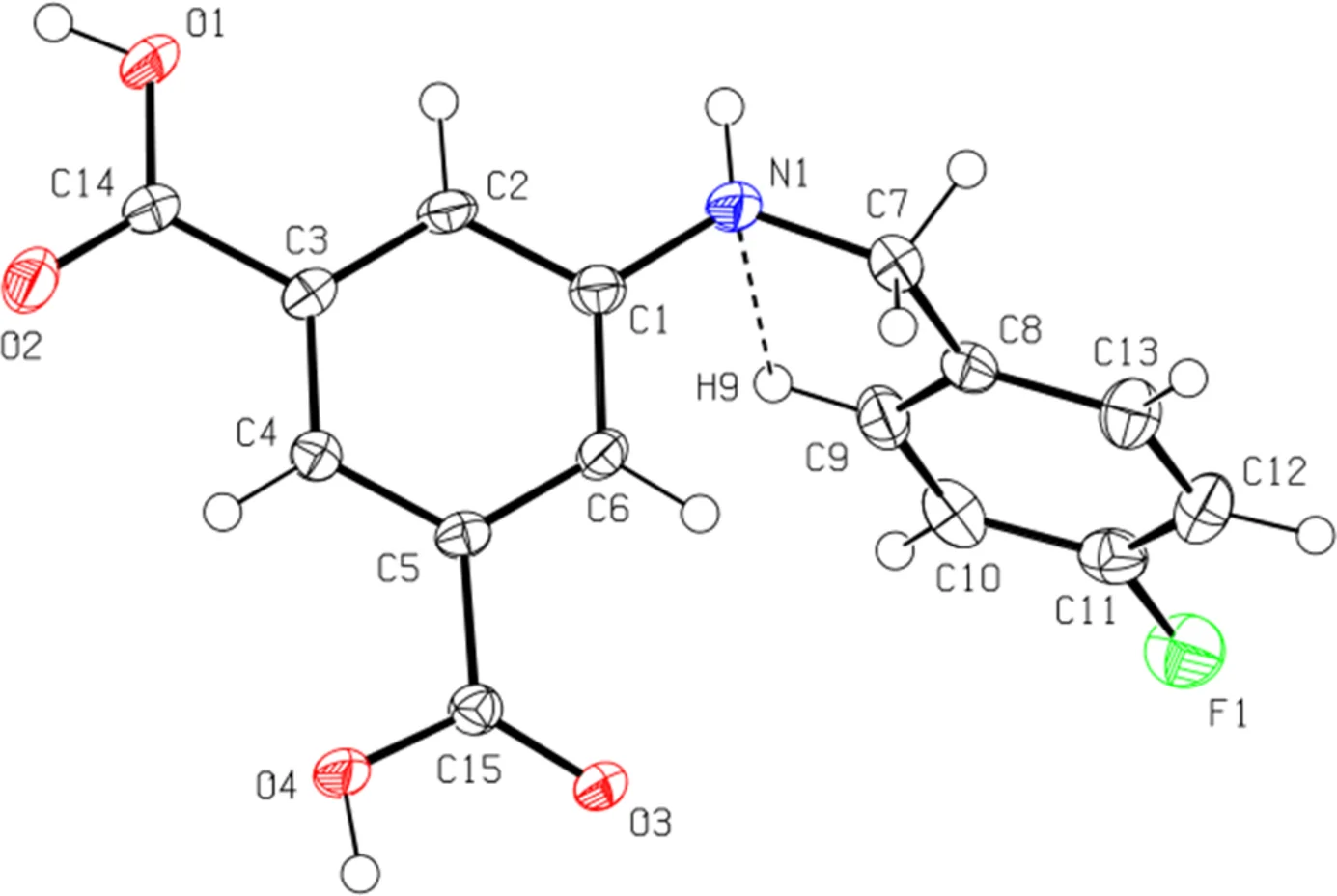
The mol­ecular structure of (I) with displacement ellipsoids drawn at the 50% probability level.

**Figure 2:**
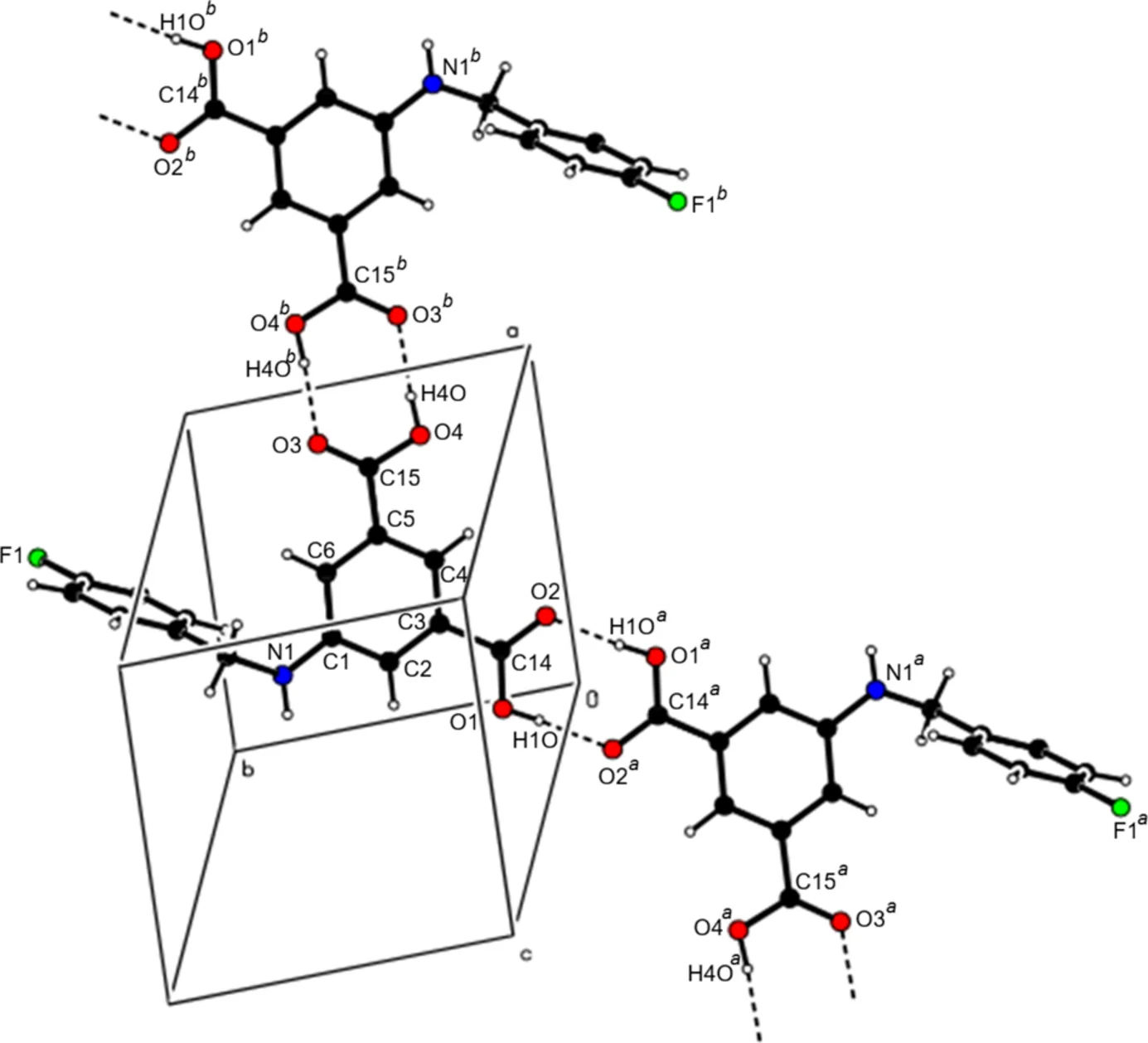
A partial view of the 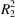
(8) motif dimers formed by pairwise O—H⋯O hydrogen bonds in the extended structure. Symmetry codes: (a) −*x*, −*y*, −*z*; (b) −*x* + 2, −*y* + 1, −*z*.

**Figure 3:**
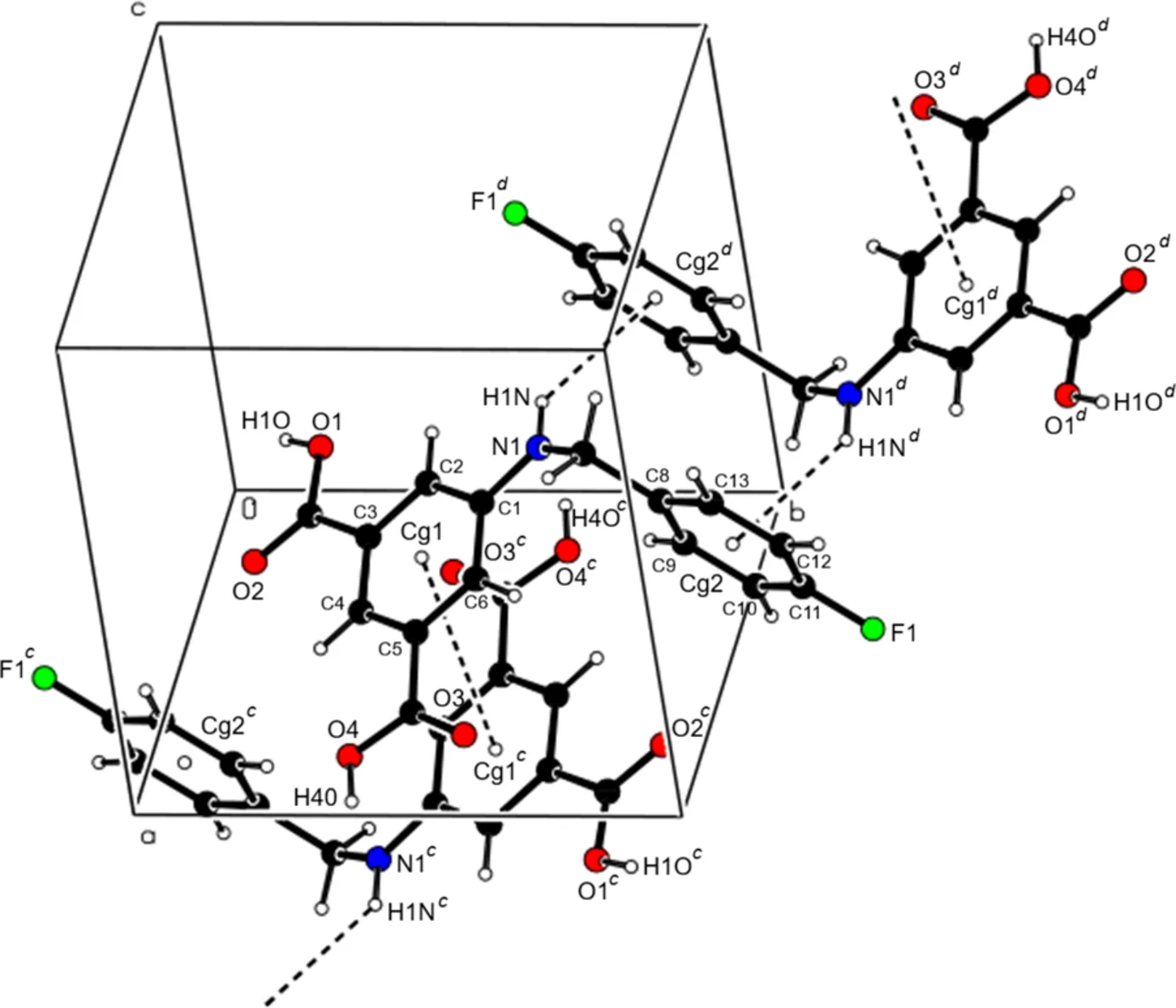
A partial view of the N—H⋯π and π–π inter­actions in the extended structure. Symmetry codes: (c) −*x* + 1, −*y* + 1, −*z*; (d) −*x* + 1, −*y* + 2, −*z* + 1.

**Figure 4:**
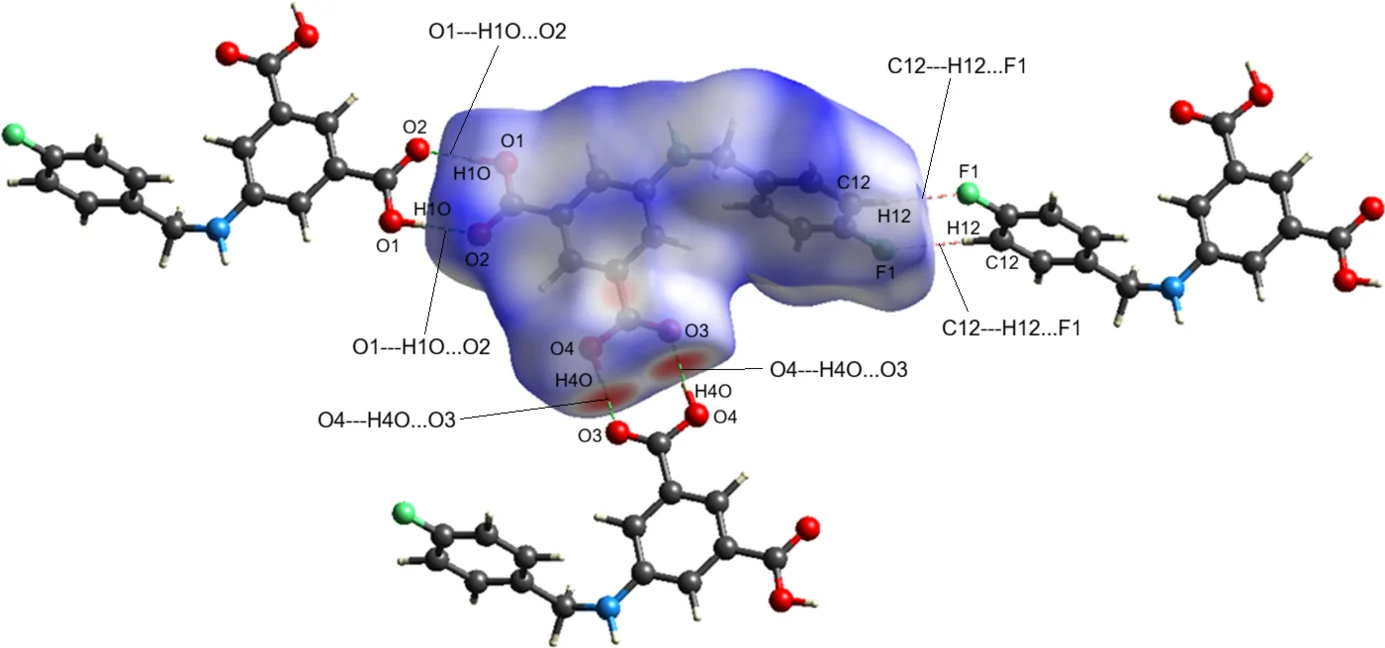
Three-dimensional Hirshfeld surface of (I) mapped over *d*_norm_.

**Figure 5:**
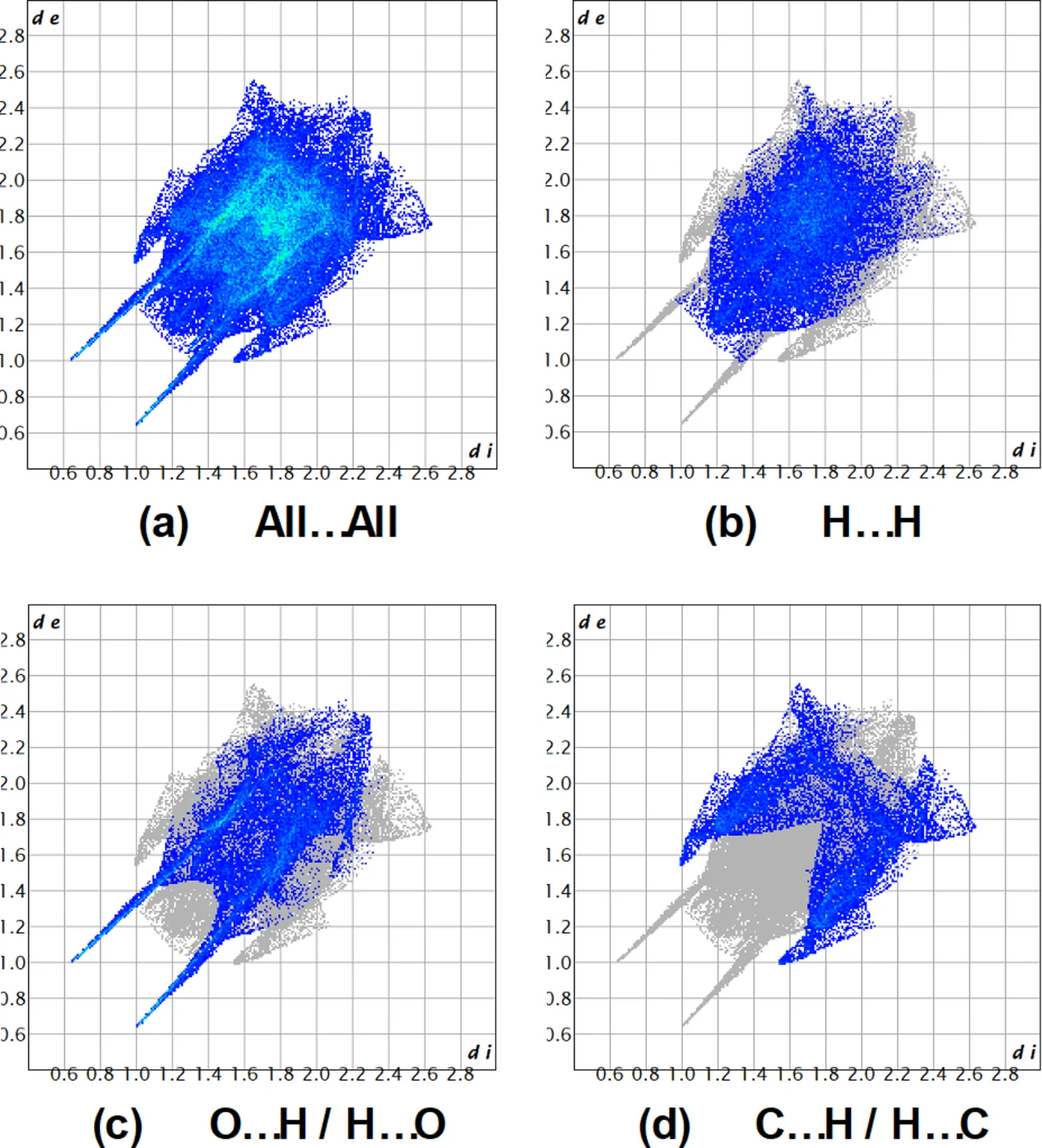
The two-dimensional fingerprint plots for (I) showing (*a*) all inter­actions, and (*b*)–(*d*) the different contact types.

**Table 1:** Hydrogen-bond geometry (Å, °) *Cg*2 is the centroid of the C8–C13 benzene ring.

*D*—H⋯*A*	*D*—H	H⋯*A*	*D*⋯*A*	*D*—H⋯*A*
C9—H9⋯N1	0.96 (1)	2.52 (1)	2.890 (2)	103 (1)
O1—H1*O*⋯O2^i^	0.86 (1)	1.77 (1)	2.6326 (16)	177 (2)
O4—H4*O*⋯O3^ii^	0.87 (1)	1.79 (1)	2.6518 (15)	176 (2)
N1—H1*N*⋯*Cg*2^iii^	0.87 (1)	2.71 (2)	3.4071 (17)	139 (2)
C12—H12⋯F1^iv^	0.96 (1)	2.46 (1)	3.2661 (19)	142 (1)
C12—H12⋯O3^v^	0.96 (1)	2.65 (2)	3.298 (2)	126 (1)

Symmetry codes: (i) 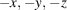; (ii) 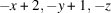; (iii) 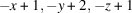; (iv) 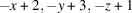; (v) 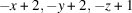.

**Table 2:** Experimental details

Crystal data	
Chemical formula	C_15_H_12_FNO_4_
*M*_r_	289.26
Crystal system, space group	Triclinic, *P* 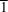
Temperature (K)	150
*a*, *b*, *c* (Å)	8.4674 (9), 9.2193 (11), 9.2749 (11)
α, β, γ (°)	102.838 (4), 96.181 (4), 108.027 (4)
*V* (Å^3^)	659.01 (13)
*Z*	2
Radiation type	Mo *K*α
μ (mm^−1^)	0.12
Crystal size (mm)	0.35 × 0.30 × 0.21

Data collection	
Diffractometer	Bruker APEXII CCD
Absorption correction	Multi-scan (*SADABS*; Krause *et al.*, 2015 ▸)
*T*_min_, *T*_max_	0.954, 0.966
No. of measured, independent and observed [*I* > 2σ(*I*)] reflections	8198, 2722, 2050
*R*_int_	0.036
(sin θ/λ)_max_ (Å^−1^)	0.627

Refinement	
*R*[*F*^2^ > 2σ(*F*^2^)], *wR*(*F*^2^), *S*	0.039, 0.102, 1.05
No. of reflections	2722
No. of parameters	236
No. of restraints	12
H-atom treatment	Only H-atom coordinates refined
Δρ_max_, Δρ_min_ (e Å^−3^)	0.24, −0.26

Computer programs: *APEX4* and *SAINT* (Bruker, 2012 ▸), *SHELXT* (Sheldrick, 2015*a* ▸), *SHELXL2014* (Sheldrick, 2015*b* ▸), *ORTEP-3 for Windows* (Farrugia, 2012 ▸) and *PLATON* (Spek, 2020 ▸).

## supplementary crystallographic information

### 5-{[(4-Fluorophenyl)methyl]amino}benzene-1,3-dicarboxylic acid Crystal data

**Table:**

C_15_H_12_FNO_4_	*Z* = 2
*M**_r_* = 289.26	*F*(000) = 300
Triclinic, *P*1	*D*_x_ = 1.458 Mg m^−^^3^
*a* = 8.4674 (9) Å	Mo *K*α radiation, λ = 0.71073 Å
*b* = 9.2193 (11) Å	Cell parameters from 2691 reflections
*c* = 9.2749 (11) Å	θ = 2.3–26.4°
α = 102.838 (4)°	µ = 0.12 mm^−^^1^
β = 96.181 (4)°	*T* = 150 K
γ = 108.027 (4)°	Prism, yellow
*V* = 659.01 (13) Å^3^	0.35 × 0.30 × 0.21 mm

### 5-{[(4-Fluorophenyl)methyl]amino}benzene-1,3-dicarboxylic acid Data collection

**Table:**

Bruker APEXII CCD diffractometer	2050 reflections with *I* > 2σ(*I*)
φ and ω scans	*R*_int_ = 0.036
Absorption correction: multi-scan (SADABS; Krause *et al.*, 2015)	θ_max_ = 26.5°, θ_min_ = 2.3°
*T*_min_ = 0.954, *T*_max_ = 0.966	*h* = −10→9
8198 measured reflections	*k* = −10→11
2722 independent reflections	*l* = −9→11

### 5-{[(4-Fluorophenyl)methyl]amino}benzene-1,3-dicarboxylic acid Refinement

**Table:**

Refinement on *F*^2^	Primary atom site location: structure-invariant direct methods
Least-squares matrix: full	Secondary atom site location: difference Fourier map
*R*[*F*^2^ > 2σ(*F*^2^)] = 0.039	Hydrogen site location: difference Fourier map
*wR*(*F*^2^) = 0.102	Only H-atom coordinates refined
*S* = 1.05	*w* = 1/[σ^2^(*F*_o_^2^) + (0.0457*P*)^2^ + 0.1305*P*] where *P* = (*F*_o_^2^ + 2*F*_c_^2^)/3
2722 reflections	(Δ/σ)_max_ < 0.001
236 parameters	Δρ_max_ = 0.24 e Å^−^^3^
12 restraints	Δρ_min_ = −0.26 e Å^−^^3^

### 5-{[(4-Fluorophenyl)methyl]amino}benzene-1,3-dicarboxylic acid Special details

**Table:**

Geometry. All esds (except the esd in the dihedral angle between two l.s. planes) are estimated using the full covariance matrix. The cell esds are taken into account individually in the estimation of esds in distances, angles and torsion angles; correlations between esds in cell parameters are only used when they are defined by crystal symmetry. An approximate (isotropic) treatment of cell esds is used for estimating esds involving l.s. planes.

### 5-{[(4-Fluorophenyl)methyl]amino}benzene-1,3-dicarboxylic acid Fractional atomic coordinates and isotropic or equivalent isotropic displacement parameters (Å^2^)

**Table:**

	*x*	*y*	*z*	*U*_iso_*/*U*_eq_	
F1	0.90425 (13)	1.37822 (11)	0.33763 (12)	0.0396 (3)	
O1	0.06367 (14)	0.18670 (14)	0.13695 (15)	0.0393 (3)	
H1O	−0.021 (2)	0.1019 (17)	0.093 (2)	0.059*	
O2	0.20181 (15)	0.07050 (14)	−0.01296 (16)	0.0469 (4)	
O3	0.92015 (12)	0.60399 (12)	0.11820 (12)	0.0242 (3)	
O4	0.79097 (13)	0.35250 (12)	−0.01900 (13)	0.0272 (3)	
H4O	0.8879 (15)	0.370 (2)	−0.048 (2)	0.041*	
N1	0.49379 (17)	0.70687 (15)	0.43757 (16)	0.0280 (3)	
H1N	0.4006 (16)	0.700 (2)	0.470 (2)	0.043 (6)*	
C1	0.49554 (18)	0.58645 (17)	0.32082 (17)	0.0210 (3)	
C2	0.34918 (19)	0.45235 (17)	0.25993 (18)	0.0213 (3)	
H2	0.2474 (14)	0.4463 (18)	0.2968 (17)	0.024 (4)*	
C3	0.35070 (18)	0.32787 (17)	0.14517 (17)	0.0201 (3)	
C4	0.49641 (18)	0.33361 (17)	0.08617 (17)	0.0195 (3)	
H4	0.4986 (18)	0.2491 (14)	0.0081 (14)	0.021 (4)*	
C5	0.63955 (17)	0.46746 (16)	0.14245 (16)	0.0182 (3)	
C6	0.63946 (18)	0.59342 (17)	0.25752 (17)	0.0200 (3)	
H6	0.7376 (14)	0.6865 (13)	0.2898 (16)	0.017 (4)*	
C7	0.6451 (2)	0.82871 (18)	0.53084 (18)	0.0255 (4)	
H7A	0.7382 (16)	0.7843 (17)	0.5426 (18)	0.027 (4)*	
H7B	0.623 (2)	0.8617 (19)	0.6336 (12)	0.029 (4)*	
C8	0.71148 (19)	0.97471 (17)	0.47556 (17)	0.0227 (4)	
C9	0.6414 (2)	0.98566 (18)	0.33749 (18)	0.0269 (4)	
H9	0.5464 (16)	0.8975 (15)	0.2762 (17)	0.034 (5)*	
C10	0.7055 (2)	1.12091 (19)	0.28966 (19)	0.0294 (4)	
H10	0.660 (2)	1.128 (2)	0.1941 (14)	0.039 (5)*	
C11	0.8393 (2)	1.24435 (18)	0.38301 (19)	0.0271 (4)	
C12	0.9112 (2)	1.23975 (19)	0.5208 (2)	0.0306 (4)	
H12	0.9998 (18)	1.3310 (15)	0.5851 (18)	0.042 (5)*	
C13	0.8465 (2)	1.10370 (19)	0.5663 (2)	0.0295 (4)	
H13	0.8931 (19)	1.0945 (19)	0.6609 (13)	0.029 (4)*	
C14	0.19642 (19)	0.18422 (18)	0.08348 (18)	0.0242 (4)	
C15	0.79660 (18)	0.48153 (17)	0.08001 (17)	0.0199 (3)	

### 5-{[(4-Fluorophenyl)methyl]amino}benzene-1,3-dicarboxylic acid Atomic displacement parameters (Å^2^)

**Table:**

	*U*^11^	*U*^22^	*U*^33^	*U*^12^	*U*^13^	*U*^23^
F1	0.0492 (6)	0.0294 (5)	0.0471 (7)	0.0152 (5)	0.0171 (5)	0.0177 (5)
O1	0.0181 (6)	0.0352 (7)	0.0475 (8)	−0.0046 (5)	0.0140 (6)	−0.0065 (6)
O2	0.0265 (7)	0.0311 (7)	0.0608 (9)	−0.0037 (5)	0.0170 (6)	−0.0162 (6)
O3	0.0163 (5)	0.0229 (5)	0.0279 (6)	0.0021 (4)	0.0077 (5)	0.0014 (5)
O4	0.0176 (6)	0.0246 (6)	0.0345 (7)	0.0044 (5)	0.0115 (5)	−0.0007 (5)
N1	0.0193 (7)	0.0247 (7)	0.0337 (8)	0.0037 (6)	0.0118 (6)	−0.0019 (6)
C1	0.0198 (8)	0.0203 (7)	0.0237 (8)	0.0071 (6)	0.0070 (6)	0.0062 (6)
C2	0.0158 (8)	0.0234 (8)	0.0263 (8)	0.0059 (6)	0.0096 (6)	0.0082 (6)
C3	0.0158 (7)	0.0199 (7)	0.0235 (8)	0.0034 (6)	0.0041 (6)	0.0077 (6)
C4	0.0189 (8)	0.0188 (7)	0.0204 (8)	0.0062 (6)	0.0048 (6)	0.0041 (6)
C5	0.0152 (7)	0.0211 (7)	0.0203 (8)	0.0068 (6)	0.0044 (6)	0.0083 (6)
C6	0.0152 (7)	0.0189 (7)	0.0235 (8)	0.0028 (6)	0.0035 (6)	0.0052 (6)
C7	0.0259 (9)	0.0250 (8)	0.0222 (8)	0.0072 (7)	0.0059 (7)	0.0012 (7)
C8	0.0219 (8)	0.0244 (8)	0.0225 (8)	0.0099 (6)	0.0076 (6)	0.0031 (6)
C9	0.0281 (9)	0.0257 (8)	0.0229 (9)	0.0095 (7)	0.0026 (7)	−0.0010 (7)
C10	0.0382 (10)	0.0307 (9)	0.0219 (9)	0.0178 (8)	0.0051 (7)	0.0043 (7)
C11	0.0317 (9)	0.0237 (8)	0.0330 (9)	0.0140 (7)	0.0148 (8)	0.0109 (7)
C12	0.0235 (9)	0.0245 (8)	0.0382 (10)	0.0046 (7)	0.0004 (7)	0.0051 (7)
C13	0.0283 (9)	0.0292 (9)	0.0287 (9)	0.0090 (7)	−0.0007 (7)	0.0079 (7)
C14	0.0172 (8)	0.0250 (8)	0.0273 (9)	0.0038 (6)	0.0074 (7)	0.0043 (7)
C15	0.0176 (8)	0.0226 (8)	0.0193 (8)	0.0067 (6)	0.0034 (6)	0.0057 (6)

### 5-{[(4-Fluorophenyl)methyl]amino}benzene-1,3-dicarboxylic acid Geometric parameters (Å, º)

**Table:**

F1—C11	1.3651 (18)	C4—H4	0.945 (9)
O1—C14	1.2809 (19)	C5—C6	1.392 (2)
O1—H1O	0.861 (10)	C5—C15	1.488 (2)
O2—C14	1.2341 (19)	C6—H6	0.953 (9)
O3—C15	1.2273 (17)	C7—C8	1.514 (2)
O4—C15	1.3156 (17)	C7—H7A	1.001 (9)
O4—H4O	0.868 (9)	C7—H7B	0.990 (9)
N1—C1	1.3728 (19)	C8—C9	1.390 (2)
N1—C7	1.4460 (19)	C8—C13	1.391 (2)
N1—H1N	0.866 (9)	C9—C10	1.387 (2)
C1—C6	1.399 (2)	C9—H9	0.961 (9)
C1—C2	1.405 (2)	C10—C11	1.371 (2)
C2—C3	1.385 (2)	C10—H10	0.952 (9)
C2—H2	0.952 (9)	C11—C12	1.371 (2)
C3—C4	1.394 (2)	C12—C13	1.382 (2)
C3—C14	1.490 (2)	C12—H12	0.958 (9)
C4—C5	1.384 (2)	C13—H13	0.955 (9)
			
C14—O1—H1O	111.3 (15)	C8—C7—H7B	108.6 (10)
C15—O4—H4O	108.2 (12)	H7A—C7—H7B	105.5 (14)
C1—N1—C7	123.58 (13)	C9—C8—C13	118.46 (15)
C1—N1—H1N	119.1 (13)	C9—C8—C7	122.41 (14)
C7—N1—H1N	116.0 (13)	C13—C8—C7	119.12 (14)
N1—C1—C6	121.89 (13)	C10—C9—C8	121.07 (15)
N1—C1—C2	120.03 (13)	C10—C9—H9	120.6 (10)
C6—C1—C2	118.07 (14)	C8—C9—H9	118.3 (10)
C3—C2—C1	120.61 (14)	C11—C10—C9	118.13 (15)
C3—C2—H2	119.2 (9)	C11—C10—H10	120.4 (11)
C1—C2—H2	120.2 (9)	C9—C10—H10	121.5 (11)
C2—C3—C4	120.87 (13)	F1—C11—C12	118.15 (15)
C2—C3—C14	120.43 (13)	F1—C11—C10	118.95 (15)
C4—C3—C14	118.69 (13)	C12—C11—C10	122.90 (16)
C5—C4—C3	118.82 (13)	C11—C12—C13	118.20 (15)
C5—C4—H4	119.8 (9)	C11—C12—H12	120.2 (11)
C3—C4—H4	121.4 (9)	C13—C12—H12	121.5 (11)
C4—C5—C6	120.82 (13)	C12—C13—C8	121.23 (16)
C4—C5—C15	120.91 (13)	C12—C13—H13	121.3 (10)
C6—C5—C15	118.26 (13)	C8—C13—H13	117.5 (10)
C5—C6—C1	120.73 (13)	O2—C14—O1	123.59 (14)
C5—C6—H6	118.9 (9)	O2—C14—C3	119.88 (14)
C1—C6—H6	120.3 (9)	O1—C14—C3	116.53 (13)
N1—C7—C8	115.64 (13)	O3—C15—O4	123.33 (13)
N1—C7—H7A	110.0 (9)	O3—C15—C5	122.30 (13)
C8—C7—H7A	107.8 (9)	O4—C15—C5	114.37 (12)
N1—C7—H7B	108.8 (10)		
			
C7—N1—C1—C6	−16.8 (2)	C7—C8—C9—C10	−179.75 (14)
C7—N1—C1—C2	164.21 (15)	C8—C9—C10—C11	−0.5 (2)
N1—C1—C2—C3	−178.04 (14)	C9—C10—C11—F1	179.82 (14)
C6—C1—C2—C3	2.9 (2)	C9—C10—C11—C12	−0.3 (2)
C1—C2—C3—C4	−0.9 (2)	F1—C11—C12—C13	−179.42 (14)
C1—C2—C3—C14	178.51 (14)	C10—C11—C12—C13	0.7 (3)
C2—C3—C4—C5	−1.1 (2)	C11—C12—C13—C8	−0.3 (3)
C14—C3—C4—C5	179.50 (14)	C9—C8—C13—C12	−0.4 (2)
C3—C4—C5—C6	1.0 (2)	C7—C8—C13—C12	−179.85 (15)
C3—C4—C5—C15	−178.08 (13)	C2—C3—C14—O2	−176.06 (16)
C4—C5—C6—C1	1.1 (2)	C4—C3—C14—O2	3.3 (2)
C15—C5—C6—C1	−179.77 (13)	C2—C3—C14—O1	3.2 (2)
N1—C1—C6—C5	177.92 (14)	C4—C3—C14—O1	−177.37 (14)
C2—C1—C6—C5	−3.1 (2)	C4—C5—C15—O3	173.38 (14)
C1—N1—C7—C8	89.38 (19)	C6—C5—C15—O3	−5.7 (2)
N1—C7—C8—C9	−5.7 (2)	C4—C5—C15—O4	−6.4 (2)
N1—C7—C8—C13	173.68 (14)	C6—C5—C15—O4	174.48 (13)
C13—C8—C9—C10	0.8 (2)		

### 5-{[(4-Fluorophenyl)methyl]amino}benzene-1,3-dicarboxylic acid Hydrogen-bond geometry (Å, º)

*Cg*1 and *Cg*2 are the centroids of the C1–C6 and C8–C13 benzene rings, respectively.

**Table:**

*D*—H···*A*	*D*—H	H···*A*	*D*···*A*	*D*—H···*A*
C9—H9···N1	0.96 (1)	2.52 (1)	2.890 (2)	103 (1)
O1—H1*O*···O2^i^	0.86 (1)	1.77 (1)	2.6326 (16)	177 (2)
O4—H4*O*···O3^ii^	0.87 (1)	1.79 (1)	2.6518 (15)	176 (2)
N1—H1*N*···Cg2^iii^	0.87 (1)	2.71 (2)	3.4071 (17)	139 (2)
C12—H12···F1^iv^	0.96 (1)	2.46 (1)	3.2661 (19)	142 (1)
C12—H12···O3^v^	0.96 (1)	2.65 (2)	3.298 (2)	126 (1)

Symmetry codes: (i) −*x*, −*y*, −*z*; (ii) −*x*+2, −*y*+1, −*z*; (iii) −*x*+1, −*y*+2, −*z*+1; (iv) −*x*+2, −*y*+3, −*z*+1; (v) −*x*+2, −*y*+2, −*z*+1.
